# Editorial: Diabetes Augmentation on Vascular Disease

**DOI:** 10.3389/fcvm.2021.810822

**Published:** 2021-12-08

**Authors:** Godfrey S. Getz, Catherine A. Reardon, Jan Boren

**Affiliations:** ^1^Department of Pathology, University of Chicago, Chicago, IL, United States; ^2^Ben May Department, University of Chicago, Chicago, IL, United States; ^3^Department of Molecular and Clinical Medicine, University of Gothenburg, Gothenburg, Sweden

**Keywords:** diabetes, cardiovascular disease, hyperglycemia, macrophage, RAGE (receptor for advanced glycation end products), postprandial glucose

Although the collection entitled Diabetes Augmentation on Vascular Disease articulates many views on which additional research and information is needed, there is universal agreement that diabetes augments the frequency of cardiovascular disease. Diabetes is an extremely common and complex disorder with a long history, being first described as polyuria (induced by the excretion of urinary sugar) in patients more than 3,500 years ago. Diabetes is the Greek word meaning “siphon.” The year 2021 is the centenary of the discovery of insulin by Banting and Best. It is anticipated that if current trends continue, by the year 2050 one in three individuals will have diabetes with the concomitant burden of vascular disease.

Diabetes enhances both macrovascular diseases, the focus of this collection, and microvascular disease (i.e., retinopathy and glomerulopathy), which is not addressed here. Diabetes attenuates the relative protective effects of cardiovascular disease in females. As pointed out by Goldberg, citing analysis of national data bases by Gregg et al. (1), the rate of vascular complications of diabetes declined significantly from 1990 to 2010 for myocardial infarction, stroke, lower limb amputation, with the greatest decline occurring in the myocardial infarction group. While myocardial infarction had the highest complication rate in 1990, by 2010 it was equivalent to stroke in the diabetic population group. This occurred in the face of increasing prevalence of diabetes over the two decades. Myocardial infarctions, stroke and amputation events reflect atherosclerosis of the coronary arteries, carotid arteries, and femoral arteries respectively. In each case the clinical complication rate is much higher for individuals with diabetes than those without diabetes. The decline in rates reflects improved treatment of hyperglycemia, dyslipidemia, and hypertension, which are common risk factors in both groups and cessation of smoking. The dyslipidemia typical of type 2 diabetes is characterized by modest if any rise in LDL cholesterol, an increase in small dense LDL, hypertriglyceridemia, and low HDL cholesterol. Even with treatment of the dyslipidemia there remains a substantial risk of atherosclerotic cardiovascular disease (ASCVD), which appears to be attributable to the plasma accumulation of triglyceride rich remnant lipoproteins containing excess quantities of apoC3 produced by the diabetic liver (2). The overproduction of apoC3 is probably a manifestation of the liver's resistance to insulin signaling. ApoC3 is an inhibitor of lipoprotein lipase leading to sustained hypertriglyceridemia when elevated. Type 2 diabetes is frequently accompanied by obesity, as an independent risk factor for the development of cardiovascular disease. Enlarged visceral adipose depots are inflamed which accounts for the insulin resistance of the metabolic syndrome that accompanies type 2 diabetes. This complex interaction between obesity, diabetes and cardiovascular disease is discussed in the comprehensive review by Chait and den Hartigh.

Hyperglycemia is the *sine qua non* of diabetes and is thought to be a risk factor for the development of ASCVD. Both diabetes and ASCVD are chronic disorders that maintain a prolonged prodrome before the manifestation of clinically recognizable disease. The review by Nahmias et al. discusses five long term clinical trials aimed at controlling hyperglycemia on ASCVD using HbA1c as a monitor of efficacy of glucose control. Only in one study is there a suggestive reduction in ASCVD related to control of hyperglycemia. Subset analysis suggests that the early initiation of diabetes treatment is most likely to be efficacious.

HbA1c measures the average plasma glucose over a 3-month interval. But it does not account for the short-term fluctuations of plasma glucose which is an important aspect of glucose homeostasis. The postprandial spikes in blood sugar (PBG) is the topic of the review by Hanssen et al. Although hyperglycemia is associated with increased ASCVD in diabetes, as mentioned above the control of the hyperglycemia is not accompanied by a reduction in the risk of cardiovascular disease —the so called “glucose paradox.” However, PBG is much better correlated with ASCVD than is HbA1c. This intermittent hyperglycemia is associated with activation of neutrophils that promotes monocytosis and thrombocytosis, both of which are related to ASCVD. In addition, hyperglycemia is associated with the increased formation of advanced glycosylation end products (AGE), which are recognized by the receptor RAGE. This receptor also recognizes non-AGE proinflammatory ligands such as S100A8/A9, S100B and HMBG1. High glucose levels promote the secretion of S100A8/A9 from neutrophils and it is postulated that its stimulation of RAGE on the common lymphoid precursor ultimately accounts for the increase in monocytes and neutrophils.

The AGE/RAGE system is involved in many inflammatory disorders as summarized by Egaña-Gorroño et al. These include atherosclerosis, peripheral vascular disease, and myocardial infarction, pathologies increased by diabetes, and obesity. RAGE is a cell surface receptor whose cytoplasmic tail interacts with diaphanous1, which is necessary for its RAGE signaling. Its extracellular portion may be released by matrix metalloproteinase and ADAM10 into the plasma. The released soluble RAGE (sRAGE) may bind RAGE ligands to attenuate RAGE signaling. That this system is significantly involved in atherosclerosis is indicated by its reduction with the expression of sRAGE or by RAGE knockout in preclinical models of atherosclerosis.

A review of the comparative composition of coronary plaques of diabetic and non-diabetic subjects reveals that the former has increased plaque burden, with increased lesional necrosis, increased macrophages and T cells and more healed plaque ruptures (3). In this collection Kanter et al. have assessed the pivotal role of monocytes and macrophages as plausible basis for the augmented ASCVD of diabetes. In diabetic cardiovascular disease, there is evidence for increased monocyte recruitment into the lesions, increased inflammatory activation of macrophages, altered macrophage lipid metabolism particularly reduced cholesterol efflux, increased cell death and decreased efferocytosis, which could contribute to the larger necrotic core. These phenotypic changes in macrophages may promote lesion progression and hinder lesion regression.

In summary, there is much yet to learn about how diabetes augments ASCVD and the development of therapeutic approaches to minimize its deleterious effects. This said, it remains essential to minimize the risk factors (hyperlipidemia, hyperglycemia, obesity, and hypertension) on an ongoing basis. To this should be added the consumption of a prudent diet.

## Author Contributions

GG, CR, and JB served as editors for the Research Topic. GG and CR wrote and edited the manuscript. JB edited the manuscript. All authors contributed to the article and approved the submitted version.

## Conflict of Interest

The authors declare that the research was conducted in the absence of any commercial or financial relationships that could be construed as a potential conflict of interest.

## Publisher's Note

All claims expressed in this article are solely those of the authors and do not necessarily represent those of their affiliated organizations, or those of the publisher, the editors and the reviewers. Any product that may be evaluated in this article, or claim that may be made by its manufacturer, is not guaranteed or endorsed by the publisher.
